# Enhanced Hydrogen Storage Kinetics of Nanocrystalline and Amorphous Mg_2_Ni-type Alloy by Melt Spinning

**DOI:** 10.3390/ma4010274

**Published:** 2011-01-18

**Authors:** Yang-Huan Zhang, Bao-Wei Li, Hui-Ping Ren, Xia Li, Yan Qi, Dong-Liang Zhao

**Affiliations:** 1 Elected State Key Laboratory, Inner Mongolia University of Science and Technology, Baotou 014010, China; E-Mails: lbaowei@126.com (B.-W.L.); renhp555@yahoo.com (H.-P.R.); Lixia-82@163.com (X.L.); 2 Department of Functional Material Research, Central Iron and Steel Research Institute, Beijing 100081, China; E-Mails: janqi_bb@163.com (Y.Q.); dlzhao@sina.com (D.-L.Z.)

**Keywords:** Mg_2_Ni-type alloy, melt spinning, hydrogen storage kinetics

## Abstract

Mg_2_Ni-type Mg_2_Ni_1−x_Co_x_ (x = 0, 0.1, 0.2, 0.3, 0.4) alloys were fabricated by melt spinning technique. The structures of the as-spun alloys were characterized by X-ray diffraction (XRD) and transmission electron microscopy (TEM). The hydrogen absorption and desorption kinetics of the alloys were measured by an automatically controlled Sieverts apparatus. The electrochemical hydrogen storage kinetics of the as-spun alloys was tested by an automatic galvanostatic system. The results show that the as-spun (x = 0.1) alloy exhibits a typical nanocrystalline structure, while the as-spun (x = 0.4) alloy displays a nanocrystalline and amorphous structure, confirming that the substitution of Co for Ni notably intensifies the glass forming ability of the Mg_2_Ni-type alloy. The melt spinning treatment notably improves the hydriding and dehydriding kinetics as well as the high rate discharge ability (HRD) of the alloys. With an increase in the spinning rate from 0 (as-cast is defined as spinning rate of 0 m/s) to 30 m/s, the hydrogen absorption saturation ratio ($R_{5}^{a}$) of the (x = 0.4) alloy increases from 77.1 to 93.5%, the hydrogen desorption ratio ($R_{20}^{\text{d}}$) from 54.5 to 70.2%, the hydrogen diffusion coefficient (*D*) from 0.75 × 10^−11^ to 3.88 × 10^−11^ cm^2^/s and the limiting current density *I_L_* from 150.9 to 887.4 mA/g.

## 1. Introduction

Among the known alloys with a potential use in hydrogen storage, Mg and Mg-based metallic hydrides are considered to be more promising materials for hydrogen storage because of their major advantages such as low specific weight, low cost and high hydrogen capacity, e.g., 7.6 wt % for MgH_2_, 3.6 wt % for Mg_2_NiH_4_ [1]. However, these kinds of hydrides suffer from high thermodynamic stability, resulting in sluggish hydriding/dehydriding kinetics which makes them still far from practical applications. During the recent years, a variety of attempts, involving mechanical alloying (MA) [2], melt spinning [3], spark plasma sintering [4], GPa hydrogen pressure method [5], hydriding combustion synthesis [6], surface modification [7], adding catalysts [8], alloying with other elements [9], *etc*., have been developed and employed to ameliorate the kinetics of Mg-based metallic hydrides. It is documented that some amorphous Mg-based alloys prepared by mechanical alloying can electrochemically absorb and desorb large amount of hydrogen at room temperature compared to their polycrystalline counterparts [10]. Their enhanced kinetics may be ascribed to the disordered character of the amorphous structure that provides the numerous desirable sites for electrochemical hydrogen storage. High energy ball-milling is regarded as a quite powerful method for the fabrication of nanocrystalline and amorphous Mg and Mg-based alloys. In particular, it is the most appropriate method to solubilize particular elements into MgH_2_ or Mg_2_NiH_4_ above the thermodynamic equilibrium limit, thus facilitating the destabilization of MgH_2_ or Mg_2_NiH_4_ [11]. However, the milled Mg_2_Ni-type alloy electrodes exhibit extremely poor electrochemical cycle stability owing to the disappearance of the metastable structures formed by ball milling during the multiple electrochemical charging and discharging cycles [12], which is an insurmountable barrier for its practical application as electrode material.

On the contrary, the melt-spinning treatment may inhibit the sharp degradation of the hydrogen absorbing and desorbing cyclic characteristics of Mg-based compounds [13]. Furthermore, the alloys, possessing the nanocrystalline and amorphous structure produced by the melt-spinning method exhibit excellent initial electrochemical hydrogen storage characteristics similar to those of the alloys fabricated by the MA process. Tanaka *et al*. [14] prepared the Mg_85_Ni_10_La_5_ hydrogen storage alloy with a nanostructure by melt spinning to obtain reversible absorption and desorption amount of about 5 wt % hydrogen at temperatures as low as 200 °C in moderate time periods. Spassov e*t al.* [15] have prepared Mg_2_ (Ni, Y) hydrogen storage alloy with the composition of Mg_63_Ni_30_Y_7_ by a rapid solidification process, exhibiting a maximum hydrogen absorption capacity of about 3.0 wt %. In addition, the melt-spun Mg_2_ (Ni, Y) alloys have demonstrated an enhanced hydrogenation kinetics compared to those of the conventionally prepared polycrystalline Mg_2_Ni alloys, to be comparable to that of the nanocrystalline ball-milled Mg_2_Ni.

In the present work, Mg-Ni-based Mg_2_Ni_1−x_Co_x_ (x = 0–0.4) nanocrystalline and amorphous alloys have been synthesized by melt-spinning technology. Moreover, the effects of the spinning rate on the structures and hydrogen storage kinetics of the alloys have been investigated.

## 2. Results and Discussion

### 2.1. Microstructures

**Figure 1:**
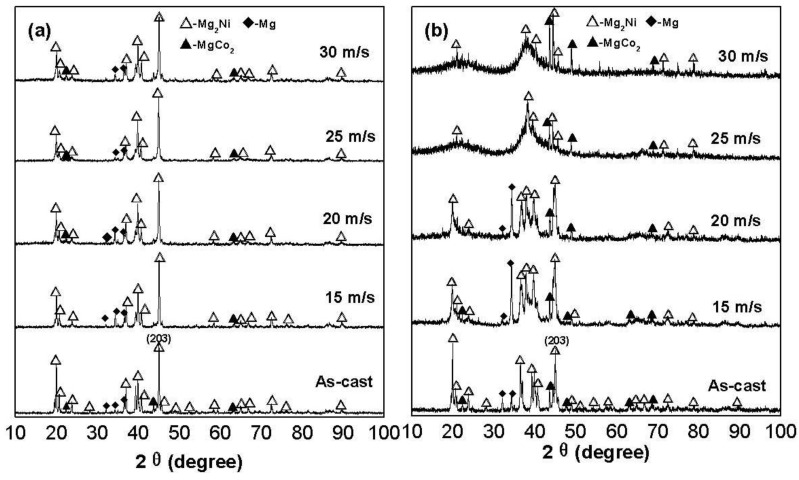
XRD profiles of the as-cast and spun alloys: (**a**) Co_0.1_ alloy; (**b**) Co_0.4_ alloy.

The X-ray diffraction (XRD) profiles of the as-cast and spun alloys are shown in Figure 1. It reveals that the as-cast and spun Co_0.1_ and Co_0.4_ alloys have a multiphase structure, comprising of a major phase Mg_2_Ni and secondary phases Mg and MgCo_2_. The melt spinning treatment engenders an unapparent impact on the structure of the Co_0.1_ alloy, while it causes a visible change of the structure of the Co_0.4_ alloy. As the spinning rate reaches 25 m/s, the Co_0.4_ alloy exhibits an obvious amorphous structure. Therefore, it may be surmised that the replacement of Ni by Co facilitates the glass formation in the Mg_2_Ni-type alloy. Two possibilities may be considered as the reasons for the above result. Firstly, the addition of a third element to Mg-Ni or Mg-Cu alloys significantly facilitates the glass-formation [16,17]. Secondly, the glass forming ability of an alloy is closely related to the difference of the atomic radii in the alloy. The higher difference of the atomic radii enhances the glass forming ability [18]. Therefore, the larger atomic radius of Co compared to that of Ni facilitates the glass-formation. Listed in Table 1 are the lattice parameters, cell volumes and full width at half maximum (FWHM) values of the main diffraction peaks of the as-cast and spun Co_0.1_ and Co_0.4_ alloys, which were calculated by using Jade 6.0 software. It is clearly viewable from Table 1 that the melt spinning causes a notably increase in the FWHM values of the main diffraction peaks of the alloys, which is doubtlessly attributed to the refined grains and the stored strain in the grains originated by the melt spinning. It is derived in Table 1 that the increase of the Co content induces not only a visible increase in the FWHM values of the main diffraction peaks of the as-spun alloys but also an evident enlargement in the lattice parameters and cell volume of the alloys, to be attributed to the larger atomic radius of Co than Ni. Based on the FWHM values of the broad diffraction peak (203) in Figure 1, the grain sizes <*D_hkl_*> (nm) of the as-cast and spun Co_0.1_ and Co_0.4_ alloys are calculated using Scherrer’s equation, also listed in Table 1. The grain sizes of the as-spun alloys are in a range of 16 to 45 nm, basically consistent with results reported by Friedlmeier *et al*. [19].

**Table 1:** Lattice parameters, cell volumes, full width at half maximum (FWHM) values and grain sizes of the as-cast and spun Co_0.1_ and Co_0.4_ alloys.

Quenching rate (m/s)	FWHM values		Grain sizes		Lattice parameters and cell Volume					
	2θ (45.14°)		D_203_ (nm)		a (nm)		c (nm)		V (nm^3^)	
	Co_0.1_	Co_0.4_	Co_0.1_	Co_0.4_	Co_0.1_	Co_0.4_	Co_0.1_	Co_0.4_	Co_0.1_	Co_0.4_
0	0.118	0.150	72	57	0.5210	0.5220	1.3244	1.3312	0.3113	0.3142
15	0.191	0.449	45	19	0.5210	0.5224	1.3251	1.3318	0.3115	0.3148
20	0.287	0.548	30	16	0.5210	0.5226	1.3258	1.3320	0.3117	0.3150
25	0.310	—	27	—	0.5211	—	1.3265	—	0.3118	—
30	0.408	—	21	—	0.5211	—	1.3287	—	0.3124	—

**Figure 2:**
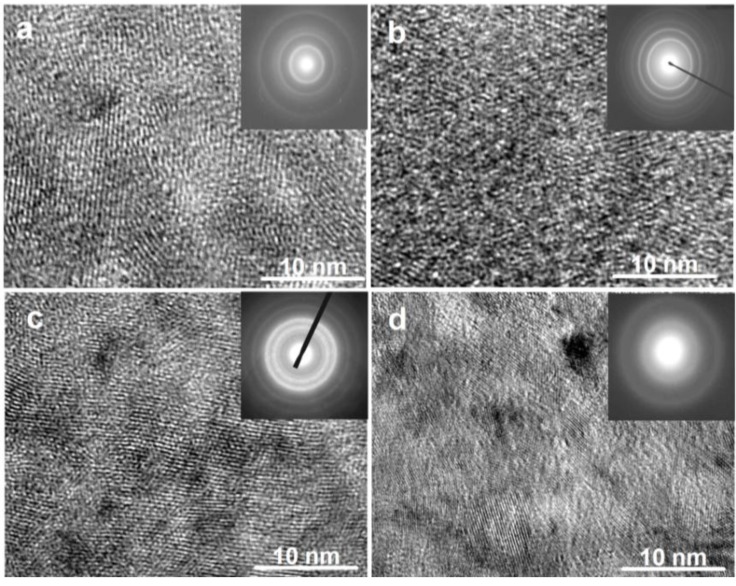
High resolution transmission electron microscope (HRTEM) micrographs and electron diffraction (ED) patterns of the as-spun alloys: (**a**) as-spun (15 m/s) Co_0.1_ alloy; (**b**) as-spun (30 m/s) Co_0.1_ alloy; (**c**) as-spun (15 m/s) Co_0.4_ alloy; (**d**) As-spun (30 m/s) Co_0.4_ alloy.

The TEM micrographs and electron diffraction (ED) patterns of the as-spun Co_0.1_ and Co_0.4_ alloys are shown in Figure 2. It is seen that the as-spun (15 m/s and 30 m/s) Co_0.1_ alloy displays a complete nanocrystalline structure, and its ED pattern appears as sharp multi-haloes, corresponding to a crystalline structure. The as-spun (15 m/s) Co_0.4_ alloy also exhibits a nanocrystalline structure, but when the spinning rate reaches 30 m/s, an amorphous phase is clearly visible in the as-spun Co_0.4_ alloy. Its electron diffraction pattern consists of broad and dull halo, confirming the presence of an amorphous structure, which agrees very well with the XRD observation shown in Figure 1.

### 2.2. Thermal Stability and Crystallization

**Figure 3:**
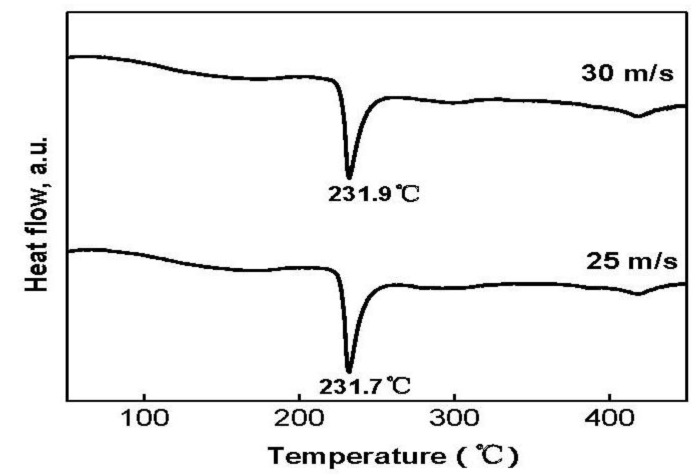
DSC profiles of Co_0.4_ alloy spun at 25 and 30 m/s.

In order to examine the thermal stability and the crystallization of the as-spun nanocrystalline and amorphous alloys, DSC analysis was conducted. The resulting profiles shown in Figure 3 reveal that during heating the alloys crystallize completely, and the crystallization process of the Co_0.4_ alloy consists of two steps. The first crystallization reaction at about 232 °C is connected with a sharp exothermic DSC peak, followed by a smaller and wider peak (418 °C) corresponding to a second crystallization reaction. It is proved that the first sharper peak corresponds to the crystallization (ordering) of the amorphous into nanocrystalline Mg_2_Ni [20].

### 2.3. Hydriding and Dehydriding Kinetics

The hydrogen absorption was carried out under 1.5 MPa hydrogen pressure (in fact, this is the initial pressure of hydriding process) at 200 °C, and hydrogen desorption at a pressure of 1 × 10^−4^ MPa at 200 °C.

**Figure 4:**
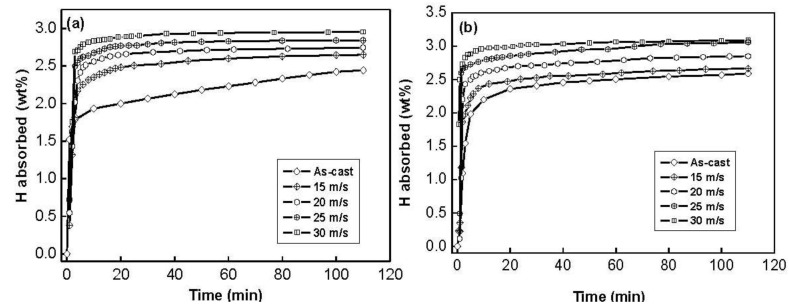
Hydrogen absorption kinetic curves of the as-cast and spun alloys: (**a**) Co_0.1_ alloy; **(b)** Co_0.4_ alloy.

The hydrogen absorption kinetic curves of the as-cast and spun Co_0.1_ and Co_0.4_ alloys are depicted in Figure 4. It is quite evident that the hydrogen absorption capacity and kinetics of all the as-spun nanocrystalline Mg_2_Ni-type alloys studied are superior to those of the as-cast ones. It can be seen in Figure 4 that the hydrogen absorption kinetics of the as-spun alloys is extremely fast so that the alloys absorb more than 85% of their hydrogen capacities within the first 5 min.

**Figure 5:**
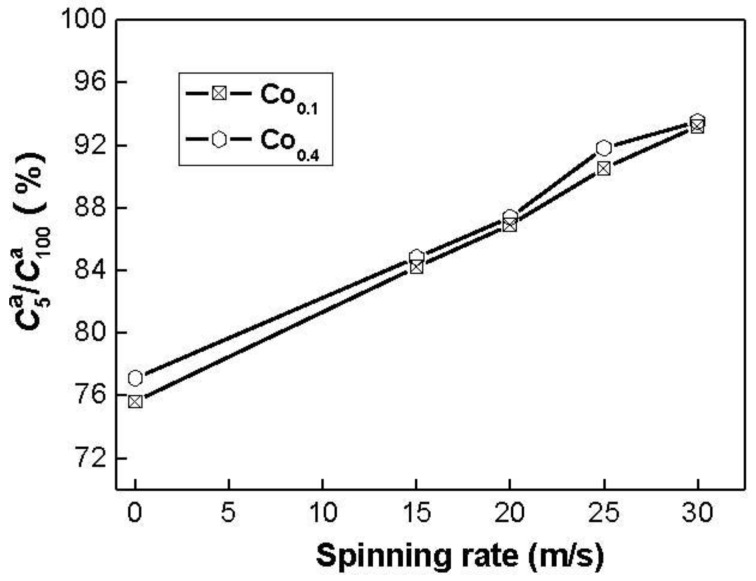
Evolution of the hydrogen absorption saturation ratio ($R_{5}^{a}$) of the alloys with the spinning rate.

The hydrogen absorption kinetics of the alloy is signified by hydriding saturation ratio ($R_{\text{t}}^{a}$), being defined as $R_{t}^{a}=C_{t}^{a}/C_{100}^{a}\times 100\%$, where $C_{100}^{a}$ and $C_{t}^{a}$ are hydrogen absorption capacities in the times of 100 min and *t* min, respectively. Apparently, for a fixed time *t*, a larger saturation ratio $R_{t}^{a}$ means better hydrogen absorption kinetics. It is reasonable to take the $C_{100}^{a}$ value as the saturated hydrogen absorption capacity of the alloy because the experimental result indicates that the $C_{100}^{a}$ value is more than 95% of the saturated hydrogen absorption capacity for all the alloys. The hydrogen absorption saturation ratio ($R_{t}^{a}$) (*t* = 5) of the alloys as a function of the spinning rate is presented in Figure 5.

The figure reveals that the $R_{5}^{a}$ values of the alloys notably increase with rising spinning rate. With an increase in the spinning rate from 0 (as-casts is defined as spinning rate of 0 m/s) to 30 m/s, the $R_{5}^{a}$ value increases from 75.6 to 93.2% for the Co_0.1_ alloy. This variable behavior may be ascribed to the structural change caused by the melt spinning. The improved hydrogen absorption kinetics can be explained with the enhanced hydrogen diffusivity in the amorphous and nanocrystalline microstructures as the amorphous phase around the nanocrystalline leads to an easier access of hydrogen to the nanograins, avoiding the long-range diffusion of hydrogen through an already formed hydride, which is often the slowest stage of absorption. Upon refining the microstructure, a lot of new crystallites and grain boundaries evolve, which may act as fast diffusion paths for hydrogen absorption [21].

**Figure 6:**
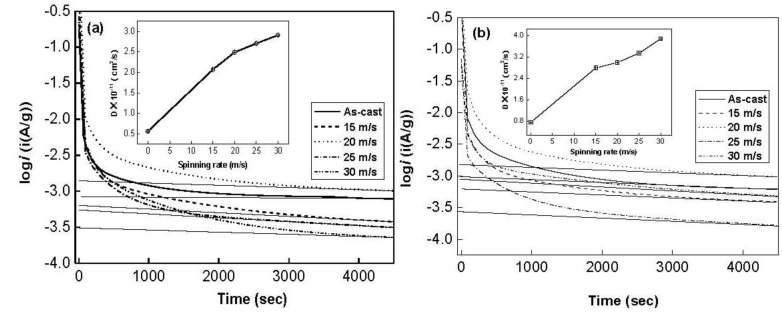
Semilogarithmic curves of anodic current *vs.* time responses of the alloys: (**a**) Co_0.1_ alloy; (**b**) Co_0.4_ alloy.

In order to reveal the mechanism of the melt spinning improving hydrogen absorption kinetics of the alloy, it is evidently necessary to investigate the influences of the melt spinning on the H diffusion ability in the alloy. The H diffusion coefficients in the as-cast and spun alloys were measured using the potential step technique. A potential step of +500 mV *versus* the stabilized open circuit potential of the fully charged electrode was applied and the decrease in discharge current was monitored as a function of time. Figure 6 shows the semilogarithmic curves of anodic current *versus* working duration of the Co_0.1_ and Co_0.4_ alloys. The diffusion coefficient *D* of the hydrogen atoms in the bulk of the alloy can be calculated through the slope of the linear region of the corresponding plots according to following formulae [22].

(1) $$\log i=\log\left(\pm \frac{6FD}{da^{2}}(C_{0}-C_{s})\right)-\frac{\pi^{2}}{2.303}\frac{D}{a^{2}}t$$

(2) $$D=-\frac{2.303a^{2}}{\pi^{2}}\frac{d\log i}{dt}$$

where *i* is the diffusion current density (A/g), *D* is the hydrogen diffusion coefficient (cm^2^/s), *C_0_* is the initial hydrogen concentration in the bulk of the alloy (mol/cm^3^), *C_s_* is the hydrogen concentration on the surface of the alloy particles (mol/cm^3^), *a* is the alloy particle radius (cm), *d* is the density of the hydrogen storage alloy (g/cm^3^), *t* is the discharge time (s), respectively. In Equation (2), $\frac{d\log i}{dt}$ is the slope of the linear region of the semilogarithmic curves of anodic current *versus* working duration, which can easily be obtained by an origin 75 software. *a* is the alloy particle radius, supposing *a =* 15 μm. Thus, the hydrogen diffusion coefficient *D* can easily be calculated. The *D* values calculated by Equation (2) are also illustrated in Figure 6. It indicates that an increase in the spinning rate turns out a growth in *D* value. As the spinning rate rises from 0 to 30 m/s, the D value increases from 5.62 × 10^−12^ to 2.91 × 10^−11^ cm^2^/s for the Co_0.1_ alloy, and from 7.49 × 10^−12^ to 3.88 × 10^−11^ cm^2^/s for Co_0.4_ alloy, which conforms very well to the result obtained by Niu *et al*. [23]. The benefaction of the melt spinning on the hydrogen absorption kinetics of the alloy is attributed to the increased cell volume and the refined grain caused by the melt spinning since the grain boundary possesses the largest hydrogen absorption capacity [24]. The above-mentioned results clarify that H diffusion ability in the alloy is a crucial factor of the hydrogen absorption kinetics of the alloy. Spassov *et al*. [25] also confirmed that the melt spinning could significantly improve the hydrogen absorption performance of Mg-based alloy, and obtains the maximum hydrogen capacity of 4.0 wt % H for the as-spun Mg_75_Ni_20_Mm_5_ (Mm = Ce, La-rich mischmetal) alloy.

**Figure 7:**
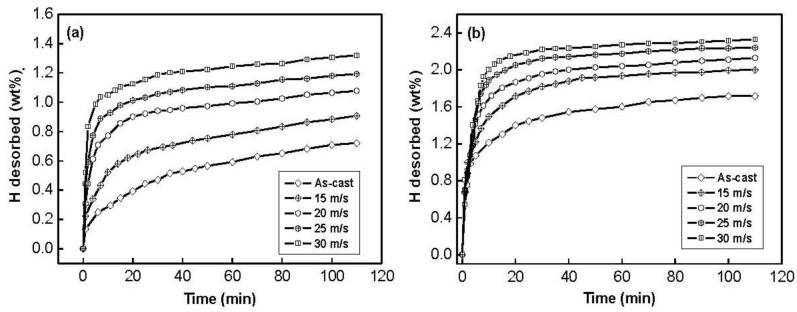
Hydrogen desorption kinetic curves of the alloys: (**a**) Co_0.1_ alloy; (**b**) Co_0.4_ alloy.

The hydrogen desorption kinetic curves of the as-cast and spun Co_0.1_ and Co_0.4_ alloys are exhibited in Figure 7. The hydrogen desorption process of the Co_0.1_ and Co_0.4_ alloys displays an evident feature, very fast initial hydrogen desorption, followed by a slack increase in the amount of hydrogen desorbed.

**Figure 8:**
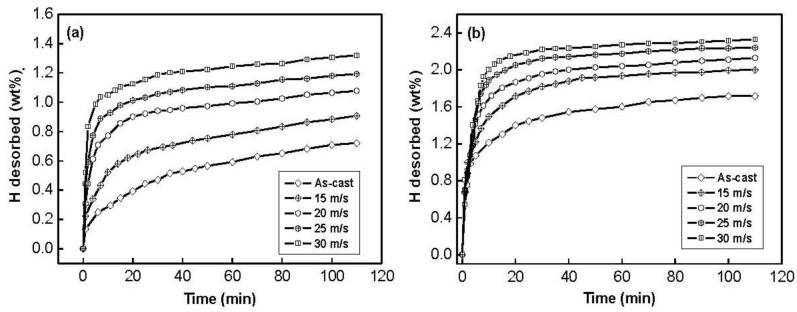
Evolution of the hydrogen desorption ratio ($R_{20}^{d}$) of the alloys with the spinning rate.

Similarly, the hydrogen desorption kinetics of the alloy is indicated by hydrogen desorption ratio ($R_{\text{t}}^{d}$), being defined as $R_{\text{t}}^{d}=C_{t}^{d}/C_{100}^{a}\times 100\%$, where $C_{100}^{a}$ is the hydrogen absorption capacity in 100 min and $C_{t}^{d}$ is the hydrogen desorption capacity in the time of *t* min, respectively. The spinning rate dependence of the hydrogen desorption ratio ($R_{20}^{d}$) (*t* = 20) of the Co_0.1_ and Co_0.4_ alloys is illustrated in Figure 8. It can be seen in Figure 8 that the melt spinning treatment remarkably enhances the $R_{20}^{d}$ values of the alloys, suggesting that melt spinning facilitates hydrogen desorption of Mg_2_Ni-type alloy. With the increase in the spinning rate from 0 to 30 m/s, the $R_{20}^{d}$ value increases from 13.2 to 38.1% for the Co_0.1_ alloy, and from 54.5 to 70.2% for the Co_0.4_ alloy. The improved hydrogen desorption kinetics is mostly associated with the change of the structure of the alloy induced by the melt spinning. It is well known that when crystalline materials are spun, they become at least partially disordered.

At same time, some crystal defects such as dislocations, stacking faults and grain boundaries are introduced. As a result, a large amount of internal energy would be stored and would lead to non-stabilization of the lattice, yielding the nanocrystalline or even an amorphous phase. Niu *et al*. [23] clarified that the introduction of defects, disordering and internal strain gives rise to increasing hydriding/dehydriding rates and capacity. It was reported [21] that the hydrogen absorbing and desorbing rates of Mg-based alloys were strongly enhanced by refinement of the grains. As noted, the internal strain increases with rising spinning rate. The higher the spinning rate, the more defects are introduced into the Mg_2_Ni alloy. Furthermore, the grain size of the alloy notably decreases with increasing spinning rate. Hence, it is understandable that the hydrogen desorption ratio ($R_{20}^{d}$) of the alloy markedly increases with rising spinning rate. It is noteworthy that, for a fixed spinning rate, the $R_{20}^{d}$ value of the Co_0.4_ alloy is much larger than that of the Co_0.1_ alloy, suggesting that increasing Co content facilitates hydrogen desorption. The increased hydrogen desorption kinetics by Co substitution is ascribed to two reasons. On the one hand, the substitution of Co for Ni notably intensifies the glass forming ability of Mg_2_Ni-type alloy because amorphous Mg_2_Ni shows an excellent hydrogen desorption capability. On the other hand, such substitution decreases the stability of the hydride and makes the desorption reaction easier [26].

### 2.4. Electrochemical Hydrogen Storage Kinetics

**Figure 9:**
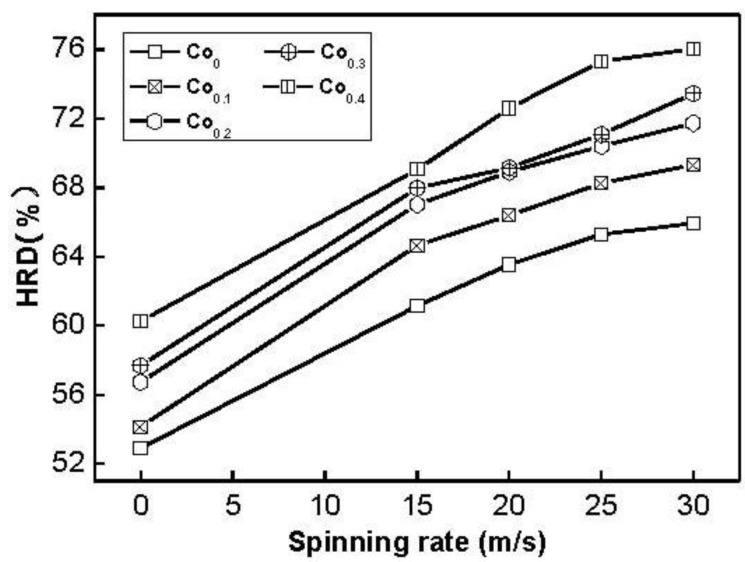
Evolution of the high rate discharge ability (HRD) of the alloys with the spinning rate.

It is quite important to restrain rapid decrease of discharge capacity even at a high charge/discharge current density for the practical application of the hydride electrode in Ni–MH battery. The electrochemical hydrogen storage kinetics of the alloy is characterized by its high rate dischargeability (HRD), being calculated according to following formula: HRD = C_100,max_/C_20,max_ × 100%, where C_100,max_ and C_20,max_ are the maximum discharge capacities of the alloy electrode charged–discharged at the current densities of 100 and 20 mA/g, respectively. The HRD values of the alloys as a function of spinning rate are exhibited in Figure 9. It is evident that the HRD values of all the alloys increase with increasing spinning rate. As the spinning rate increases from 0 to 30 m/s, the HRD value rises from 54.2 to 69.3% for the Co_0.1_ alloy, and from 60.3 to 76.0% for the Co_0.4_ alloy. High rate discharge ability (HRD) is a kinetic performance of hydrogen absorbing/desorbing of the alloy electrode, basically depending on the charge transfer at the alloy-electrolyte interface the hydrogen diffusion process from the interior of the bulk to the surface of alloy particle [27]. The melt spinning treatment visibly enhances the HRD values of the alloys, to be attributed to a positive action of the melt spinning on the hydrogen diffusion in the alloy as shown in Figure 6.

**Figure 10:**
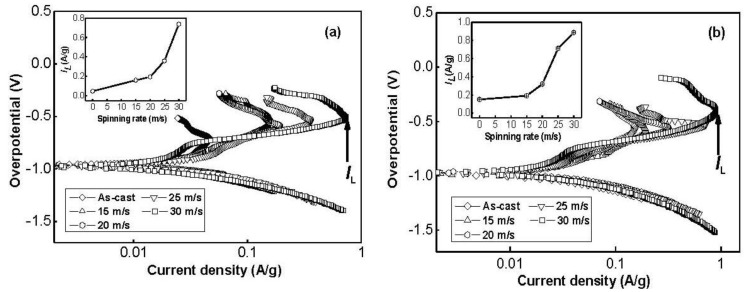
Tafel polarization curves of the as-cast and spun alloy electrodes at the 50% depth of discharge (DOD): (**a**) Co_0.1_ alloy; (**b**) Co_0.4_ alloy.

To determine the kinetics of hydrogen absorption/desorption, Tafel polarization measurements were carried out on the experimental alloy electrodes. Figure 10 depicts the Tafel polarization curves of the as-cast and spun Co_0.1_ and Co_0.4_ alloy electrodes at the 50% depth of discharge (DOD). It is quite evident that, in all cases, the anodic current densities increase to a limiting value, then decrease. The existence of a limiting current density, *I_L_*, indicates that an oxidation reaction took place on the surface of the alloy electrode, and the generated oxidation product resists further penetration of hydrogen atoms [28]. The decrease of the anodic charge current density on cycling implies that charging is becoming more difficult. Thus, the limiting current density, *I_L_*, can be seen as the critical passivation current density. It is viewable in Figure 10 that *I_L_* values of the alloys notably increase with rising spinning rate. With an increase in the spinning rate from 0 to 30 m/s, *I_L_* value increases from 46.7 to 737.3 mA/g for the Co_0.1_ alloy, and from 150.9 to 887.4 mA/g for the Co_0.4_ alloy. Ratnakumar *et al*. [29] and Liu *et al*. [30] presumed that the limiting current can be related to and is mainly controlled by the solid state diffusion of hydrogen in metal-hydride electrodes. Hence, the positive impact of the melt spinning on the *I_L_* value can be ascribed to the change of the structure of the alloy induced by the melt spinning. Furthermore, Figure 10 displays an interesting phenomenon that the corresponding peak potential of the limiting current apparently shifts to positive direction with increasing spinning rate, indicating an enhanced excellent ability of antioxidation or anticorrosion by the melt spinning.

**Figure 11:**
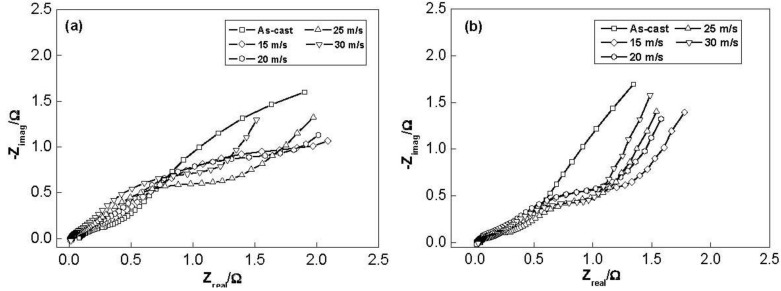
Electrochemical impedance spectra (EIS) of the alloy electrodes at the 50% depth of discharge (DOD): (**a**) Co_0.1_ alloy; (**b**) Co_0.4_ alloy.

The electrochemical impedance spectra (EIS) of the as-cast and spun Co_0.1_ and Co_0.4_ alloy electrodes at 50% DOD are shown in Figure 11. It is viewable that each EIS spectrum contains two semicircles followed by a straight line. Kuriyama *et al*. [31] considered that the smaller semicircle in the high frequency region is attributed to the contact resistance between the alloy powder and the conductive material, while the larger semicircle in the low frequency region is attributed to the charge-transfer resistance on the alloy surface. The linear response at low frequencies is indicative of hydrogen diffusion in the bulk alloy. Hence, the electrode kinetics of the as-cast and spun alloys are dominated a mixed rate-determining process. It can be seen in Figure 11 that the radius of the large semicircle in the low frequency for the Co_0.1_ and Co_0.4_ alloy clearly decreases with increasing spinning rate, implying that the refined grain by the melt spinning facilitates charge-transfer of the alloy electrode. Based on the above mentioned results, it can be concluded that a suitable microstructure is quite crucial to obtain the excellent hydrogen storage kinetics of the Mg_2_Ni-type alloy.

## 3. Experimental Section

The nominal compositions of the experimental alloys were Mg_2_Ni_1−x_Co_x_ (x = 0, 0.1, 0.2, 0.3, 0.4). For convenience, the alloys were denoted with Co content as Co_0_, Co_0.1_, Co_0.2_, Co_0.3_ and Co_0.4_, respectively. The alloy ingots were prepared by using a vacuum induction furnace in a helium atmosphere at a pressure of 0.04 MPa. A part of the as-cast alloys was re-melted and spun by melt-spinning with a rotating copper roller. The spinning rate was approximately expressed by the linear velocity of the copper roller. The spinning rates used in the experiment were15, 20, 25 and 30 m/s, respectively.

The phase structures of the as-cast and spun alloys were determined by X-ray diffraction (XRD) (D/max/2400). The diffraction, with the experimental parameters of 160 mA, 40 kV and 10°/min, respectively, was performed with CuK_α1_ radiation filtered by graphite. The effective crystal sizes were calculated from Scherrer’s formula [32].

The thin film samples of the as-spun alloys were prepared by ion etching method in order to observe the morphology with high resolution transmission electron microscopy (HRTEM) (JEM-2100F, operated at 200 kV), and also to determine the crystalline state of the samples with electron diffraction (ED). The average grain sizes of the as-spun alloys were measured by a linear intercept method on the HRTEM micrographs.

Thermal stability and crystallization of the as-spun alloys were studied by means of DSC instrument (STA449C), and the heating temperature and rate are 600 °C and 10 °C /min, respectively.

The hydrogen absorption and desorption kinetics of the alloys were measured by an automatically controlled Sieverts apparatus. Prior to measuring the hydriding and dehydriding kinetics of the alloys, several hydrogen absorbing and desorbing cycles were carried out in order to activate the materials. The hydrogen absorption was conducted at 1.5 MPa and 200 °C, and the hydrogen desorption at a pressure of 1 × 10^−4^ MPa and 200 °C.

The alloy ribbons were pulverized into fine powder of about 20 μm by mechanical milling and then mixed with carbonyl nickel powder in a weight ratio of 1:4. The mixture was cold pressed under a pressure of 35 MPa into round electrode pellets of 10 mm in diameter and total mass of about 1 g. The electrochemical characteristics of the alloy electrodes were tested by a tri-electrode open cell, consisting of a metal hydride electrode, a sintered NiOOH/Ni(OH)_2_ counter electrode and a Hg/HgO reference electrode. The electrolyte is a solution of 6 M KOH. The voltage between the negative electrode and the reference electrode was defined as the discharge voltage. In every cycle, the alloy electrode was first charged at a constant current density, and following resting for 15 min, it was discharged at the same current density to −0.500 V cut-off voltage. The environment temperature of the measurement was kept at 30 °C.

The electrochemical impedance spectra (EIS) and the Tafel polarization curves of the alloys were measured using an electrochemical workstation (PARSTAT 2273). The fresh electrodes were fully charged and then rested for 2 h up to the stabilization of the open circuit potential. The EIS spectra of the alloy electrodes were measured in the frequency range from 10 kHz to 5 mHz at 50% depth of discharge (DOD). The Tafel polarization curves were measured in the potential range of −1.2 to +1.0 V (*vs*. Hg/HgO) with a scan rate of 5 mV/s. For the potentiostatic discharge, the test electrodes in the fully charged state were discharged at 500 mV potential steps for 4500 s on electrochemical workstation (PARSTAT 2273), using the CorrWare electrochemistry corrosion software.

## 4. Conclusions

The investigation of the structures of the Mg_2_Ni_1−x_Co_x_ (x = 0, 0.1, 0.2, 0.3, 0.4) alloys indicates that a nanocrystalline and amorphous structure can be obtained in the experiment alloys by melt spinning technology. The substitution of Co for Ni facilitates the glass formation in the Mg_2_Ni-type alloy. Moreover, the amorphization degree of the alloys visibly increases with increasing spinning rate. The melt spinning significantly improves the hydrogen storage kinetics of the alloys. The hydrogen absorption saturation ratio ($R_{\text{t}}^{a}$) and hydrogen desorption ratio ($R_{\text{t}}^{d}$), as well as the high rate discharge ability (HRD), increase with rising spinning rate. The hydrogen diffusion coefficient (D), the Tafel polarization curves and the electrochemical impedance spectra (EIS) measurements show that the electrochemical kinetics notably increases with rising spinning rate.
